# Paranoia as an Antecedent and Consequence of Getting Ahead in Organizations: Time-Lagged Effects Between Paranoid Cognitions, Self-Monitoring, and Changes in Span of Control

**DOI:** 10.3389/fpsyg.2016.01446

**Published:** 2016-09-22

**Authors:** Niels Van Quaquebeke

**Affiliations:** Management, Kühne Logistics UniversityHamburg, Germany

**Keywords:** paranoia, self-monitoring, getting ahead, span of control, zero-inflated negative binomial regression, cognitions, management, career

## Abstract

A 6-month, time-lagged online survey among 441 employees in diverse industries was conducted to investigate the role paranoia plays as an antecedent and as a consequence of advancement in organizations. The background of the study is the argument that it requires active social sense-making and behavioral adaptability to advance in organizations. The present paper thus explores the extent to which employees’ paranoid cognitions—representative of a heightened albeit suspicious sense-making and behavioral adaptability—link with their advancement in organizations (operationalized as changes in afforded span of control), both as an antecedent and an outcome. Following the strategy to illuminate the process by interaction analysis, both conditions (antecedent and outcome) are examined in interaction with employees’ self-monitoring, which is considered representative of a heightened but healthy sense-making and behavioral adaptability. Results support the expected interference interaction between paranoid cognitions and self-monitoring in that each can to some degree compensate for the other in explaining employees’ organizational advancement. Reversely, changes in span of control also affected paranoid cognitions. In particular, low self-monitors, i.e., those low in adaptive sense-making, reacted with heightened paranoid cognitions when demoted. In effect, the present study is thus the first to empirically support that paranoid cognitions can be a consequence but also a prerequisite for getting ahead in organizations. Practical advice should, however, be suspended until it is better understood whether and under what circumstances paranoia may relate not only to personally getting ahead but also to an increased effectiveness for the benefit of the organization.

## Introduction

While the study of leadership effectiveness is undoubtedly important for understanding what gets organizations ahead, it is the study of leadership emergence^1^ that often strikes closer to home because it explains what type of people get ahead. It resonates with our romantic attachment to leaders (Meindl et al., 1985) and provides fuel to those wanting to become leaders themselves. Despite prevalent enchantment with becoming a leader, promotions to leadership positions might, however, also come at a price. Anecdotes describe how social dynamics can dramatically change for people once they are promoted to lead others (Sennett, 2000; Kramer, 2001).

In the present paper, I seek to shed light on both issues. Specifically, building on Kenny’s and Zaccaro’s (1983) account of leader social sensitivity and flexibility as prerequisites for leadership emergence and (Kramer’s (2001) analysis of organizational paranoia, I will focus on paranoia as an antecedent but also as a consequence of span of control. Paranoia has a rich clinical psychological tradition (American Psychiatric Association, 2000), yet to this date remains largely uninvestigated with regard to the issue of leadership (an exception is the theoretical account of Kramer and Gavrieli, 2004). Interestingly, though, in its symptomatic expression paranoia resembles self-monitoring, a trait that has already been closely linked with the issue of leadership emergence (Day et al., 2002). Given the resemblance of both concepts, I will explore both in concert to elucidate more of the underlying social additivity and sensitivity mechanism.

In sum, the current study seeks to investigate whether paranoia is not only adaptive for getting ahead in organization but also whether it may be the result of promotion and demotion processes (as evidenced in changes in afforded span of control). It thereby complements the rather popular literature with empirically driven insights (e.g., Grove, 1997; Babiak and Hare, 2007; Collins and Hansen, 2011; Ghaemi, 2011) and extends the available scientific literature on psychopathologies at work (cf. Judge et al., 2009; O’Boyle et al., 2012) with the soundly established clinical psychological concept of paranoia. As such, the present study is to my knowledge the first to empirically explore the “darker” path to and from leadership via the concept of paranoia. The present study, additionally, adds to the debate on what is state and what is trait. In particular, by looking at paranoid cognitions not only as an antecedent but also as an outcome of changes in afforded span of control, the study design follows Zaccaro’s (2007) call to investigate if and to what degree manager dispositions are malleable due to situational circumstances. This approach mirrors advances in fundamental psychology that argue that the trait/state divide may not be as clear cut as once believed (Mischel and Shoda, 1995; Fleeson, 2001; Mischel, 2004; Hudson et al., 2012) – an insight that has by and large not permeated the organizational literature so far. Third, the present research also follows another one of Zaccaro’s (2007) calls for future trait research, namely to consider meaningful patterns and integration of multiple attributes. Against this background, specifically looking at self-monitoring as a moderator of paranoia permits a more in-depth interpretation of the explanatory dynamics at play in light of hard-to-assess mediators (Spencer et al., 2005; Jacoby and Sassenberg, 2011).

In the following, I will separately delineate paranoia as an antecedent and as an outcome of span of control. Respective results will also be depicted and interpreted separately but then tied together in a discussion.

### Why Paranoia and Self-monitoring Support Getting Ahead

The conventional account argues that people’s social sense-making and adaptivity are prerequisites for career advancement because those abilities allow respective individuals to effectively navigate the political sphere of organizations (Foti and Hauenstein, 1998; e.g., Kenny and Zaccaro, 1983; Zaccaro et al., 1991; Turnley and Bolino, 2001; Yukl and Mahsud, 2010). In the following, I will first outline how self-monitoring feeds into this social sense-making and additivity, to then argue that the same dynamics also hold for paranoia, and, as such, both can be assumed to substitute for each other in their effect on getting ahead in organizations.

Self-monitoring is a trait that has been extensively studied in the organizational domain, specifically with regard to its potency for getting ahead in organizations (Day et al., 2002). Self-monitoring is defined as a consistent pattern of individual differences in the extent to which people regulate their self-presentation by tailoring their actions in accordance with immediate situational cues (Snyder, 1974; Lennox and Wolfe, 1984). The ability of high self-monitors to observe and control their appearance in social settings and interpersonal relationships is seen as an interpersonal competitive advantage in organizations, especially when it comes to career progress (for an overview see: Day et al., 2002; Day and Schleicher, 2006); with the meta-analysis of Day et al. (2002) showing a corrected correlation of 0.21 between self-monitoring and leadership emergence (in its various operationalizations, cf. Kaiser et al., 2008). The argument in a nutshell is that organizations are complex arena of social relations. Hence, those who want to advance need to be able to competently and confidently maneuver in this arena. High self-monitors are not only sensitive to the social cues around them, but also capable to adaptively move on the social parquet, and hence they advance more easily than low self-monitors. Yet, very similar arguments might also be put forward for a slightly “darker” construct: paranoia.

Paranoia has a rich tradition in clinical psychology (American Psychiatric Association, 2000), but to this date has largely been omitted from micro organizational research (an exception is the theoretical account of Kramer and Gavrieli, 2004). Paranoia is defined as a mental state characterized by a pervasive, long-standing suspiciousness and generalized mistrust of others (American Psychiatric Association, 2000). When diagnosed as a stand-alone personality disorder, the WHO’s ICD-10 (1993) lists (1) excessive sensitivity to setback and rebuffs, (2) tendency to bear grudges persistently, (3) a combative and tenacious sense of personal rights, (4) tendency to experience excessive self-importance, and (5) recurrent suspicions without justification regarding sexual fidelity of spouse of sexual partner, (6) suspiciousness and a pervasive tendency to distort experience by misconstruing the neutral or friendly action of others as hostile or contemptuous and (7) preoccupation with unsubstantiated “conspiratorial” explanations of events both immediate to the patient and the world at large. For a clinical diagnosis of a full paranoid personality disorder, three of these symptoms need to be present.

Paranoia, however, can also show in a milder and more malleable form as ‘paranoid delusions’. Content-wise corresponding ideations often relate to point 5 and 6 in the personality disorder. Thus also called persecutory delusions (Freeman, 2007). Importantly, such paranoid thoughts are not all that uncommon in the regular population (10–15% according to Freeman, 2007; more than 25% according to Verdoux and van Os, 2002) and they are not necessarily considered pathological. Affected individuals are oversensitive, easily feel slighted, and vigilantly scan the environment for clues or suggestions that may confirm their fears or preconceptions. Indeed, paranoid individuals are eager, however, somewhat irrational observers (Freeman et al., 2012). They think they are in danger and look for signs and threats of that danger. The person’s subsequent paranoid cognitions often revolve around a concern for how others might observe or try to harm him or her (Freeman et al., 2005). As a consequence, affected individuals are always on high alert and try to maneuver in what they consider a ‘smart’ way to evade or even counter the impeding attack (Fein, 1996; de Vries, 2006).

With this in mind, the argument made for self-monitoring can also be made for paranoia. Paranoid individuals also constantly scan their social environment and control and adapt their behavior toward others – albeit for different reasons than high self-monitors (Kramer, 1994). Whereas high self-monitors feel confident that they can read the social environment and proactively respond to it, paranoid individuals often feel helpless in social contexts and hence devise defensive strategies to cope with the uncertainty. Both strategies are social sense-making strategies. High self-monitors try to understand the social environment to build relationships (Kilduff et al., 2001), paranoid individuals try to understand the social environment in order not to become the target of anyone. As Kramer and Gavrieli (2004, pp. 251–252) put it “In acute sensemaking predicaments, where the costs of misguided action may be catastrophic to a leader, more effortful and mindful modes of information processing may be enormously useful. Thus, precisely *because* leaders are so willing to allocate cognitive resources to sensemaking tasks, they might be more likely to detect early and formulate response to threats that others underestimate or overlook.”

As both constructs, paranoid cognitions and self-monitoring, evoke social sense making and adaptability which has been argued to be a central prerequisite for career advancement (Foti and Hauenstein, 1998; e.g., Kenny and Zaccaro, 1983; Zaccaro et al., 1991; Turnley and Bolino, 2001; Yukl and Mahsud, 2010), and the link between self-monitoring and getting ahead in organizations has already been meta-analytically confirmed (Day et al., 2002), the following interaction hypothesis can be put forward:

*Hypothesis 1: Both paranoia and self-monitoring can compensate for the lack of the other in their effect on getting ahead in organizations*.

### Why and When Getting Ahead May Inform Paranoia

Following Kramer’s (2001) account of organizational paranoia, the second objective of the present paper is to investigate whether changes in span of control also affect people’s paranoid cognitions. Indeed, social dynamics change once people are promoted to lead others or demoted to lead fewer. For example, being awarded more span of control often would be understood as a sign of afforded trust, while losing span of control may prompt respective individuals to think that others are out to sabotage them and, as a demotion suggests, have already partly succeeded (Kramer, 1996, 1998). Because any significant organizational process will be met with a sense-making process (Jaques, 1953; Weick, 1995), both kinds of incidents will spur sense-making questions in individuals trying to answer why this has happened to them (Diamond et al., 2004).

Given people’s propensity for self-serving biases and protecting their self-esteem (Baumeister, 1998), promoted individuals will likely recognize their promoters’ goodwill and see the reason for their promotion within themselves. In contrast, demoted individuals likely identify external parties as the cause for personal ‘failure’ experiences. Fiske et al. (1996, p. 116) argue furthermore that being subjected to such potentially ill-willed external control, such as in the case of a demotion, will “make powerless people anxious by threatening basic motives for competence, control, and self-esteem.” Additional recent physiologically grounded research supports this view by showing that cortisol (an indicator of anxiety) is positively related to not feeling in control (Sherman et al., 2012). So it is in particular a demotion that may prompt paranoid thought while a promotion may reduce it.

Yet, again, promotions and demotions are organizational dynamics that call for social sense-making. In this respect, self-monitoring may once again exhibit a moderating effect by guiding respective individuals to more sensible and potentially less sinister inferences (Ferris et al., 2007). This follows studies that have, for instance, found that higher self-monitoring goes hand-in-hand with decreased social anxiety (Lennox and Wolfe, 1984, *r* = -0.18) and produces a significantly lower tendency to engage in social comparison (Gibbons and Buunk, 1999, *r* = -0.23). As such, high self-monitoring may be able to provide a (sensemaking) buffer against negative situations and thus resultant anxiety. In sum this would mean, that an individual’s cognitive downward spiral into paranoid thought as a result of a demotion may be buffered by that individual’s high self-monitoring ability. With this understanding, a second interaction hypothesis can be formulated:

Hypothesis 2: Decreases in span of control are positively related to changes in paranoid cognitions, and this effect is stronger for low self-monitors than for high self-monitors.

## Materials and Method

### Sample

Participants were recruited as part of a large clinical study in Germany that was investigating the relationship between paranoid cognitions in the general population and respective decision and safety behavior in two cross-sectional designs (reference omitted to ensure review blindness). The present study used only participants that volunteered information at both time points and could be matched for its cross-lagged analyses (see sample description below).

Invitations were sent out to all available working panelists in the WiSo online panel. The panel was set up with help of considerable third party Government funds. It is dedicated exclusively to academic research. Participants can fill out the respective questionnaires online whenever convenient for them, i.e., at home or at work. For their participation, participants received bonus points within the online panel system that they could later exchange for products that would be sending to them. As such, the WiSo panel is very similar to the US panel Amazon mTurk for which studies attest the psychometric quality (e.g., Paolacci et al., 2010; Buhrmester et al., 2011) putting such means of data collection at least on par with regular surveys (Gosling et al., 2004). To be able to answer the directionality implied in both hypotheses, all measures were collected at two time points 6 months apart.

Analyses were conducted across 441 time-matched participants that were considered sound. Data from individuals who, without a single exception, crossed items consistently at either the lowest or highest possible point on all scales at both times, T1 and T2, were excluded due to their suspicious answering behavior (cf. Meade and Craig, 2012). Furthermore, because the Global Fortune500 ranking identifies the Deutsche Post as Germany’s biggest employer (in 2011, worldwide 13th) with 418,000 employees, an additional 10 participants were excluded who indicated that their current organization size is above that number.

The sample’s mean age was 41 years (*SD* = 11.86) with women representing 55.21% of the sample. Of all participants, 42.28% had a university degree and 64.47% had completed an (additional) apprenticeship. Individuals came from a wide variety of industry sectors such as production, finance, or educational institutions as well as a wide variety of departments such as sales, customer service, or research and development. Following the reasoning that paranoid cognitions should help in improving span of control and not necessarily with getting or losing a job, participants were included if they had a job at T1 *and* T2. However, even if the criterion involved having a job either at T1 *or* T2, possibly due to the reasoning that paranoid individuals might obtain but also lose their (leadership) job faster, the ultimate sample size would increase by 32, but results would not fundamentally change. Further, because the objective of the present research is to reveal changes in span of control, people remained in the sample irrespective of whether they were actually leading other people at T1 or T2.

### Measures

Self-monitoring was assessed at T1 with the 11-item revised self-monitoring scale by Lennox and Wolfe (1984). Sample items include “In social situations, I have the ability to alter my behavior if I feel that something else is called for” and “If someone is lying to me, I usually know it at once from the person’s manner of expression.” Answers were made on a five-point Likert scale ranging from “fully disagree” to “fully agree.”

Paranoia was assessed via paranoid cognitions at T1 and T2 with the 18-item Paranoia Checklist (Freeman et al., 2005), which has been deemed appropriate when testing for paranoia symptoms in non-clinical samples (e.g., “There might be negative comments being circulated about me.”; “People would harm me if given an opportunity.”). Answers were made on a five-point Likert scale featuring the choices: “almost never,” “monthly,” “weekly,” “several times a week” and “daily.”

Getting ahead was measured via changes in afforded span of control. To this end, span of control was assessed at T1 and T2 by asking participants about how many people in their organization are considered their subordinates (directly and indirectly). Zero was the default for no span of control.

Next to gender and age, which have been shown to correlate with self-monitoring (Day et al., 2002), two additional variables were assessed as controls at T1 because of their potential link with the outcome: (1) highest education degree because it often marks an entry requirement for leadership positions, and (2) organization size because it might be easier to obtain a leadership position when there are more opportunities to lead people.

## Results

**Table 1** shows the descriptives, intercorrelations, and psychometric properties of the scales involved. Expectedly, paranoid cognitions covary (*r* = 0.6). The degree of covariation suggests around 36% stability over time. Remaining unexplained variance allows for the second analysis in which paranoid cognitions are investigated as an outcome of changes in span of control. The covariation of span of control at T1 and T2 indicates 80% stability over the 6-month time frame. However, much of the stability is due to the fact that many respondents who did not have span of control at T1 also did not have any at T2. Such a high amount of zeros needs be taken into account when deciding upon the appropriate analysis method – as will be explained in the section hereafter. With that said, some changes did occur: 11% saw an increase in span of control and 13% saw a decrease in span of control. Collectively, this led to a slight drop in the mean span of control from T1 to T2, which is also visible in the difference score that will be used for the second part of the subsequent analysis. The similarity of means and standard deviations of constructs that were measured at T1 and T2 bolsters confidence in the quality of the sample as big deviations could be taken a sign of survey faking (Moritz et al., 2012).

**Table 1:** Descriptives, intercorrelations, and internal reliabilities.

	*M*	*SD*	1	2	3	4	5	6	7	8	9
(1) Gender	1.46	0.50	(na)								
(2) Age	41.00	11.90	0.11^∗^	(na)							
(3) Education	5.04	1.84	0.01	-0.12^∗∗^	(na)						
(4) Organization size	7011	33548	-0.04	-0.05	0.03	(na)					
(5) Paranoid Cognitions T1	1.74	0.73	0.12^∗^	-0.10^∗^	-0.16^∗∗∗^	0.05	(0.95)				
(6) Paranoid Cognitions T2	1.71	0.73	0.10^∗^	-0.09	-0.12^∗^	0.06	0.60^∗∗∗^	(0.95)			
(7) Self-monitoring T1	3.47	0.54	-0.06	0.10^∗^	0.02	0.01	-0.07	-0.05	(0.83)		
(8) Span of control T1	11.83	76.89	0.01	0.04	0.00	-0.01	-0.02	0.00	0.10^∗^	(na)	
(9) Span of control T2	9.70	67.04	0.01	0.06	0.02	-0.01	-0.03	0.02	0.10^∗^	0.90^∗∗∗^	(na)
(10) Change in Span of Control from T1 to T2	-2.13	34.12	-0.01	0.04	0.04	0.00	-0.01	0.03	-0.04	-0.49^∗∗∗^	-0.06

*N = 441, ^∗^*p* < 0.05, ^∗∗^*p* < 0.01, ^∗∗∗^*p* < 0.001; Gender: 1 = female, 2 = male; two-sided; Cronbach’s alpha in brackets in the diagonal where applicable*.

In the following, I will describe the two analyses separately, i.e., (a) paranoid cognitions as an antecedent of span of control changes and (b) as a consequence of changes in span in control. Because the to be predicted outcomes are of different psychometric type, also different kinds of regression analysis need to be employed.

### Analysis of Paranoia and Self-Monitoring as Antecedents of Getting Ahead

Because the dependent variable, span of control change, is a count variable, Poisson type of regressions are advised (Gardner et al., 1995). In case of over-dispersed count data, the method of choice is a negative binomial regression as it overcomes the highly restrictive boundary conditions for Poisson regressions. In the present case concerning span of control, the variable is not only over-dispersed, but zeros (i.e., no span of control either at T1 or T2) comprise around one-third of the entries. More recent advances in statistics are able to adjust for such distributions by modeling a zero-inflation (Long, 1997). The zero-inflation model generates a second model that will be fitted simultaneously to the specified regression model to account for excess zeros so that they do not distort the regression.

Statistical tests are available to ascertain the statistical path to take (Long and Freese, 2006). For instance, in the present case, a likelihood-ratio test of the natural log of the over-dispersion coefficient returns an alpha of 1.73 (robust *SE* of 0.232; untransformed coefficient is 5.627 with a robust *SE* of 1.307), which is significantly different from 0 (χ^2^= 2538.35, *p* < 0.001); this confirms that a zero-inflated negative binomial model is preferred over an ordinary zero-inflated Poisson-model. Additionally, the Vuong-test (*z* = 1.70, *p* = 0.049) confirms that the zero-inflated negative binomial is preferred over an ordinary negative binomial regression model. Finally, robust regression can be applied to adjust for heterogeneity in the data. Hence, for the present data analysis a robust zero-inflated negative binomial regression is employed and computed with STATA. Due to the inherent count nature of the dependent variable, the ensuing analysis follows a logarithmic function, meaning that *y*-axis unit increases per *x*-unit are log increases thus requiring slightly different plotting of figures and slopes analysis.

The above entails that two models have to be considered: (1) a model predicting the change in span of control and (2) a model predicting the excessive zeroes in span of control. With regard to the first model: Negative binomial regressions cannot handle negative dependent variables. Thus instead of simply taking a difference score between T2 and T1 in span of control to assess change, the analysis predicts T2 while controlling for the effect of T1, thus essentially also predicting change between T2 and T1 through the other predictor variables in the model (Edwards, 2001). With regard to the second model: The assumption inherent in zero-inflated modeling is that a separate process creates the excessive zeros. For the present analysis, the above-described control variables were thus inserted not only in the count model but also in the inflate model. That is gender, age, education, or size of the organization could be related to not having any span of control at all. In line with good statistical practice, both models retained the control variables only if they had a significant impact and if the overall model fit saw improvement (Becker, 2005).

**FIGURE 1:**
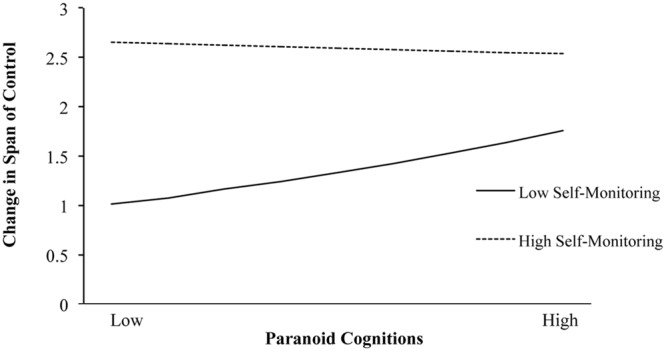
**The interactive effects of paranoid cognitions and self-monitoring on changes in span of control**.

The best-fitting model identified participants’ self-monitoring, paranoia and their interaction in the count model, with participants’ highest educational degree and gender showing as predictors in the zero-inflation model (Wald-χ^2^= 18.88, *p* < 0.001). Other potential control variables (organizational size and participant age) did not contribute significantly in the count or the zero-inflation model, nor did they interact with the focal predictors; these variables were thus omitted. The final models are shown underneath each other in **Table 2.** Please note that according with good statistical practice predictors were standardized before entering them and their interaction term (Cohen et al., 2003). The non-significant main effect between span of control at T1 and T2 confirms the inflated correlation in the regular correlation depicted in **Table 1** due to the excessive amount of zeros in the data. As expected, self-monitoring at T1 relates to a logarithmic increase in span of control. As a main effect, paranoia at T1 shows no effect on change in span of control. Most importantly, however, the main effects are qualified by the interaction between self-monitoring and paranoia. The significant interaction pattern is plotted in **Figure 1.** As can be seen, and in line with Hypothesis 1, higher paranoia explains increases in span of control under the condition of low self-monitoring [Incident Rate Ratio *(IRR)* = 1.629, *SE* = 0.270, *z* = 2.94, *p* = 0.003, *ll-CI_(95%)_ =* 1.176, *ul-CI_(95%)_* = 2.256], but not under the condition of high self-monitoring (*IRR* = 1.200, *SE* = 0.146, *z* = 1.49, *p* = 0.135, *ll-CI_(95%)_* = 0.945, *ul-CI_(95%)_* = 1.524). In other words, paranoia and self-monitoring can compensate for the lack of the other in explaining increases in span of control.

**Table 2:** A zero-inflated negative binomial regression to explain (a) the count variable changes span of control (span of control at T2 while controlling for span of control at T1) and (b) the excessive amount of zeros in the count variable.

		B	SE (robust)	z	p	ll-CI	ul-CI
Count model	Constant	0.988	0.565	2.12	0.034	0.076	1.900
	Span of control T1	0.026	0.019	1.40	0.161	0.010	0.063
	Self-monitoring T1 (SM)	0.335	0.127	2.65	0.008	0.087	0.583
	Paranoid Cognitions T1 (P)	0.126	0.095	1.33	0.183	-0.060	0.312
	Interaction (SM^∗^P)	-0.153	0.072	-2.11	0.035	-0.295	-0.011
Inflate model	Constant	4.240	1.162	3.65	0.000	1.962	6.519
	Education	-0.408	0.152	-2.69	0.007	-0.706	-0.110
	Gender	-2.261	1.016	-2.22	0.026	-4.253	-0.027

**N* = 441, lower (ll) and upper (ul) level for Confidence Intervals (CI) at 95%.*

Besides the focal count model, the additional zero-inflation model shows the significance of the participants’ highest education degree and their gender with regard to having no span of control. Corresponding with standard hiring and promotion procedures in organization, lower educational degrees expectedly go hand-in-hand with a lower chance of receiving a leadership position. Additionally, and as role congruity theory elucidates (Eagly and Karau, 2002), females have a lower likelihood of obtaining any leadership position at all.

### Analysis of Paranoia as a Consequence of Getting Ahead and Self-Monitoring

When analyzing the effects of changes in paranoid cognitions on changes in span of control in interaction with self-monitoring, a regular OLS regression is the appropriate course of analysis. Also, because of its count nature, it is now fitting to operationalize change span of control as the difference between span of control at T1 and T2 (Edwards, 2001) and entering the difference as an independent variable in the model. Note that change in paranoia (similar as change in span of control in the previous analysis) is captured by controlling for paranoia at T1 while explaining paranoia at T2.

**FIGURE 2:**
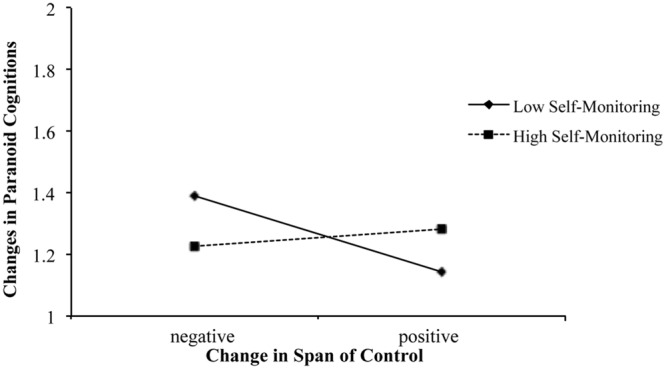
**The interactive effects of changes in span of control and self-monitoring on changes in paranoid cognitions**.

Respective results are displayed in **Table 3.** While none of the predictors beyond paranoia at T1 has a significant main effect on paranoia at T2, the interaction between change in span of control and self-monitoring is significant. The model in total explains a *R*^2^ of 0.37. The corresponding **Figure 2** shows, as expected, that changes in span of control affect high and low self-monitors differently. Specifically, simple slopes analysis (at ±1 *SD* of the moderator) reveal that low self-monitors experience a drop in paranoia with increases in span of control (*B* = -0.082, *SE* = 0.033, *t* = -2.47, *p* = 0.014, *ll-CI_(95%)_* = -0.147, *ul-CI_(95%)_* = 0.017), reversely suggesting that losing span of control can prompt paranoid cognitions. In line with Hypothesis 2, high self-monitors are not affected by changes in span of control (*B* = 0.069, *SE* = 0.038, *t* = 1.81, *p* = 0.071, *ll-CI_(95%)_* = -0.006, *ul-CI_(95%)_* = 0.144).

**Table 3:** Regression model to explain change in paranoid cognitions (paranoid cognitions at T2 controlled for paranoid cognitions at T1) by change in span of control from T1 to T2 in a linear interaction with self-monitoring.

	*B*	*SE (robust)*	*t*	*p*	*ll-CI*	*ul-CI*
Constant	0.669	0.009	7.51	0.000	0.494	0.844
Paranoid cognitions T1	0.599	0.055	10.9	0.000	0.491	0.707
Change in Span of Control from T1 to T2 (CSC)	-0.049	0.029	-1.7	0.089	-0.105	0.008
Self-Monitoring T1 (SM)	-0.006	0.026	-0.24	0.809	-0.058	0.045
Interaction (CSC^∗^SM)	0.075	0.024	3.12	0.002	0.028	0.123

**N* = 441, lower (ll) and upper level (ul) for Confidence Intervals (CI) at 95%, *R*^2^ = 0.37.*

## Discussion

The present study is one of the few that, as called for (Spain et al., 2014), empirically links clinical psychological research—as established in standard diagnostic instruments such as the World Health Organization’s (1993) ICD-10 or the American Psychiatric Association’s DSM-IV (2000)—with applied organizational research. Specifically, the present study is the first to explore the role of paranoia as an antecedent and outcome of span of control (see Kramer, 2001, for a broader account of organizational paranoia). In doing so, the present study significantly adds to previous studies that have explored psychopathologies as antecedents of getting ahead in organizations (Judge et al., 2009; O’Boyle et al., 2012). By additionally confirming an interference interaction with self-monitoring, a first step in elucidating the underlying process was taken (Spencer et al., 2005; Jacoby and Sassenberg, 2011). In particular, the nature of the interaction suggests that the active ‘ingredient’ to get ahead may be similar in paranoia and self-monitoring and, given the conceptualization of both constructs, likely revolves around the proposed mechanism of social sensemaking and adaptability.

As a further contribution, the present study is also among the first to submit that changes in span of control will have an effect on the individuals who attain them. The significant interaction with self-monitoring again suggests that social sense-making may lie at the heart of the process. As such, the present research also opens a discourse about how certain dispositions are malleable in the face of situational factors – a discourse that has been called for in applied psychology (Zaccaro, 2007) and rests on developments in fundamental personality psychology (Mischel and Shoda, 1995; Fleeson, 2001; Mischel, 2004; Hudson et al., 2012).

While the effect sizes of both interactions are not large, an important point to realize here is that it is that interaction effects are often underestimated in survey research (McClelland and Judd, 1993; Aguinis, 1995). As Evans (1985) points out as a result of his Monte Carlo analysis, interactions with as little as 1% variance accounted for should be taken seriously.

### Applied Implications

From a practical perspective, it is interesting to consider both, an individual’s paranoid cognitions as an a) antecedent and as a b) consequence of promotions (and demotions) in organization.

(a) As put forward in the paper’s introduction, the reasoning for why paranoia and self-monitoring can compensate for the lack of the other in explaining increases in span of control is that both concepts come with a heightened social sensitivity and adaptivity (“social chameleon”). This is beneficial in organizational politics not only to position oneself for positions of leadership but also to stay clear of issues that can potentially hurt one’s career (cf. Bolino and Turnley, 2003, who argue similarily with respect to impression management that different paths may be taken; Kramer, 2001). A hasty reaction to the present results might be that organizations should rid themselves of respective ‘pathological,’ i.e., paranoid, elements before they manage to rise to the top. However, such actions might throw out the baby with the bathwater. Indeed, many psychological anomalies are adequate psychological responses at a certain place and time, yet inappropriate at a different place and time (Dickinson and Eva, 2006). This is also the case for paranoid delusions (Maher, 1988; Green et al., 2011; Marr et al., 2012). Indeed, for paranoid delusions, today’s organizations might be the right place and time.

The self-monitoring literature, for instance, suggests that the social skills of high self-monitors also help them become effective (Day and Schleicher, 2006). Day et al. (2002), for instance, find in their meta-analysis a corrected *r* of 0.10 with performance. Kilduff et al. (2001) even argue that high self-monitors not only emerge as leaders but are also more effective than low self-monitors because of their ability to manage social networks. Interestingly for the context of the present study, researchers present a similar effectiveness argument for paranoid individuals. Take the already above introduced quote from Kramer and Gavrieli (2004, pp. 251–252), for instance, that “In acute sensemaking predicaments, where the costs of misguided action may be catastrophic to a leader, more effortful and mindful modes of information processing may be enormously useful. Thus, precisely *because* leaders are so willing to allocate cognitive resources to sensemaking tasks, they might be more likely to detect early and formulate response to threats that others underestimate or overlook.” Similarly, popular writers such as Collins and Hansen in their recent book “Great by choice” (2011) go beyond the social sense-making and adaptivity argument and propose that paranoid individuals are often great leaders because they permanently ask “what if” questions. Their analysis mirrors the attitude of former Intel President and CEO Grove (1997) who, by his own account, constantly worried about screwed-up products, products getting introduced prematurely, underperformance of factories or having too many factories, hiring the right people, whether morale slacks off, and about competitors that will figure out a better and cheaper way to deliver what Intel delivers. Such paranoid cognitions, Collins and Hansen (2011) argue, are particularly valuable in an increasingly uncertain world where economic crises, global competition, technological disruption and all kinds of other threats represent the rule rather than the exception. Those who are paranoid are often better prepared to meet these challenges. In that sense, it seems being paranoid could be the essence of adaptivity and thus pivotal to success (cf. evolutionary accounts: Darwin, 1859; Haselton and Nettle, 2006).

Hence, for now, I would hold off with practical advice until further research is conducted that investigates whether and under what circumstances paranoid delusions also have an effect on leadership effectiveness. Such future findings may mirror the more positive takes on paranoia as those described above, or they may unearth unwanted collaterals of paranoid leaders, such as a trickled-down anxiety among their employees, a general avoidance focus at the expense of any approach tendencies, or structuring of work relationships according to theory X (instead of theory Y) because then they seem more controllable. In any case, further research is needed before the results of the present study truly fall into place with regard to their specific implications for practice.

(b) Regarding paranoid cognitions as a consequence: It seems that the self-entrapping character of paranoia—as Kramer (2001, p. 24) put it, “Once in doubt, always in doubt”— might not always hold. As the data show while paranoid cognitions may be fueled by demotions, they can also be interrupted by increases in span of control. Notably, this effect is absent for high self-monitors, which suggests that the social sense-making capabilities inherent in these individuals provide them with some resilience against the ups-and-downs of organizations. Thus, it seems, high self-monitors are not only likely to emerge as leaders organizations (Day et al., 2002)., they also seem well equipped to deal with the uncertain consequences.

### Limitations and Research Perspectives

Naturally, the present paper is not without limitations. First, although the present study was conducted as part of a larger clinical psychological study in the general population, the recruited sample only involved people who still managed to work (see sample description). More severe forms of paranoia often do not allow people to even go outside of their home, and, indeed, maybe they would also not fill out surveys online for fear of identification. Thus, the results of the present study should only be interpreted within the boundaries of non-clinical paranoia. Indeed, it may be interesting to investigate when paranoid cognitions loose their adaptive function for individuals and become burdensome pathologies that impair social cohesion and performance (cf. Baysinger et al., 2014; Spain et al., 2014). Such designs may be explored in clinical samples where distortions in social sense-making may be modeled against the severity of experienced paranoid cognitions.

Second, getting ahead was measured via the proxy of changes in afforded span of control. While promotions and demotions with regard to personnel responsibility are straight-forward to assess and leave little room for ambiguity or self-serving biases, the concept of getting ahead in organizations is arguably much broader. Future investigations may thus turn to additional criteria of career success such as number of promotions (e.g., Kilduff and Day, 1994) or compensation packages (e.g., O’Reilly et al., 2013) to see how robust the current findings are.

Third, it seems worthwhile to explore further boundary conditions. While the present analysis was able to rule out moderators such as organization size and several demographics, the literature on organizational politics, for instance, suggests that aspects such as centralization, formalization, advancement opportunities, job autonomy, and feedback may also play a role for those that dance on the social parquet of organizations (Ferris and Kacmar, 1992; Kacmar, 1999; Blickle et al., 2012). Investigating environments where paranoid individuals as well as high self-monitors may strive versus falter seems an important further stepping stone for understanding the dynamics of social sensitivity and adaptivity at work.

Fourth and related to the above, while there is much communality between the business world in Germany and other countries, results of the Global Leadership and Organizational Behavior Effectiveness program (Brodbeck et al., 2002) assert that Germany’s corporate culture can be described as low on compassion, high on performance paired with a high power distance. It may be that this environment uniquely caters to paranoid individuals to get ahead but also reduces paranoia once people are promoted. Low power distance cultures with higher accountability toward all sides may, for instance, show reverse effects that paranoia increases the higher one is promoted in the hierarchy. The identified effects thus await intercultural confirmation.

## Conclusion

The popularity of recent studies with a psychopathological view on people at the top (e.g., Kets de Vries, 1994, 2003, 2012; Babiak and Hare, 2007; Collins and Hansen, 2011; Dutton, 2012) are an indication of our fascination with psychological disorders that enable people to get ahead in organizations. To some extent such popularity signals that the experiences of the wider public resonate with these investigations. As suggested in books such as “Snakes in Suits” (Babiak and Hare, 2007) or “A First-rate Madness: Uncovering the Links between Leadership and Mental Illness” (Ghaemi, 2011), it seems personality defects can be quite adaptive in certain environments. Or as former Intel President and CEO Grove (1997) put it more succinctly: “Only the paranoid survive.”

## Author Contributions

The author confirms being the sole contributor of this work and approved it for publication.

## Conflict of Interest Statement

The author declares that the research was conducted in the absence of any commercial or financial relationships that could be construed as a potential conflict of interest.

## Footnotes

1 Note that I follow a recent differentiation of the various types of leadership measures by Kaiser et al. (2008) in which selection for a leadership role and respective promotions that include increases in span of control are categorized under “leadership emergence.”
