# Corrigendum: Paranoia as an Antecedent and Consequence of Getting Ahead in Organizations: Time-Lagged Effects Between Paranoid Cognitions, Self-Monitoring, and Changes in Span of Control

**DOI:** 10.3389/fpsyg.2016.01796

**Published:** 2016-11-15

**Authors:** Niels Van Quaquebeke

**Affiliations:** Management, Kühne Logistics UniversityHamburg, Germany

**Keywords:** paranoia, self-monitoring, getting ahead, span of control, zero-inflated negative binomial regression, cognitions, management, career

In the first paragraph of Section-Materials and Method section and Subsection-Sample, of the original article, it says “(reference omitted to ensure review blindness).” Instead it should say “(Moritz et al., 2012; Moritz and Van Quaquebeke, 2014).”

## Conflict of interest statement

The author declares that the research was conducted in the absence of any commercial or financial relationships that could be construed as a potential conflict of interest.
